# Competition limits first-year growth and flowering of wiregrass (*Aristida beyrichiana*) at a sandhills restoration site

**DOI:** 10.1371/journal.pone.0297795

**Published:** 2024-09-03

**Authors:** Debriana T. Love, Jennifer M. Fill, April Zee, Sarah Tevlin, Héctor E. Pérez, Raelene M. Crandall

**Affiliations:** 1 School of Forest, Fisheries, and Geomatics Sciences, University of Florida, Gainesville, Florida, United States of America; 2 Department of Environmental Horticulture, University of Florida, Gainesville, Florida, United States of America; Southeastern Louisiana University, UNITED STATES OF AMERICA

## Abstract

Uncertainty in ecosystem restoration can be mitigated by information on drivers of variability in restoration outcomes, especially through experimental study. In southeastern USA pine savannas, efforts to restore the perennial bunchgrass wiregrass (*Aristida beyrichiana*) often achieve variable outcomes in the first year. Although ecotypic differentiation and competition with other native vegetation are known to influence wiregrass seedling establishment and growth, to our knowledge, no studies have examined interactions between these drivers. We experimentally quantified individual and interactive effects of competition, seed source, and soil type on wiregrass density, size, and flowering culm production in the field. We sowed seeds from dry and wet sites reciprocally into dry and wet soils and weeded half of the plots. We found that competition removal resulted in significantly larger plants and a greater proportion of flowering plants with more culms on average, regardless of seed source or soil type. Seeds sourced from a wet site resulted in more plants per plot than seeds from a dry site, which might have been influenced by the greater number of filled seeds from the wet site. After seedlings become established, competition contributes to variation in growth and reproduction. Although competition removal could help start wiregrass populations, the necessity of mitigation depends on fire management needs.

## Introduction

Ecosystem restoration is an uncertain and unpredictable process that benefits from experimental study. Identifying drivers of variability in restoration outcomes is a key step in mitigating uncertainty [1]. Restoration outcomes may be very different even when similar approaches or techniques are applied to similar sites [2]. Although possible drivers can be suggested by retrospective and comparative observational studies, experimental studies are the “gold standard” for identifying mechanisms that could cause variability in restoration trajectories and outcomes [2–4]. Experimental studies support the identification of individual and interactive effects of environmental conditions and restoration techniques, increasing the predictive power of ecosystem restoration models. Predictive models should be particularly useful during the first few years of restoration when most resources are devoted to projects [5].

Variation in restoration outcomes is evident in fire-prone grassland and savanna ecosystems that have high herbaceous plant species diversity. Reestablishment of native herbaceous vegetation is one of the most common restoration goals in degraded grasslands and savannas that have little remnant vegetation [6]. Understory plants are foundational for ecosystem resilience because of their relationship with soil communities, ecosystem processes such as fire, and animal habitat requirements [7, 8]. Because fire-prone, grassy ecosystems often have so many herbaceous species, the overall response of the understory to different restoration treatments can mask the responses of individual species. For example, [9] found that although restored sites had greater cover than the original degraded sites, survival and cover of individual species varied. Dominant or foundational species such as wiregrass, however, often have important relationships with species diversity or regulate ecosystem processes such that their successful restoration may be a priority [10, 11].

In pine savannas of the southeastern U.S., restoration goals typically include reinstating frequent, low-intensity fires by planting flammable, dominant bunchgrass species. Pine savannas are open-canopied ecosystems that have a sparse overstory of pines such as longleaf (*Pinus palustris* Mill.) and slash pines (*Pinus elliottii*), and a diverse understory of grasses, shrubs, and forbs. Located within a biodiversity hotspot [12], their extent has been drastically reduced due to continual anthropogenic pressures such as agricultural expansion, urbanization, and fire exclusion [9]. Therefore, this ecosystem is a focus of widespread restoration efforts, which commonly begin by reestablishing wiregrass (*Aristida beyrichiana*). Bunchgrasses are a prominent fuel source for spreading fires during prescribed burns [13, 14]. Wiregrass, in conjunction with other understory bunchgrasses such as dropseed (*Sporobolus junceus*) and little bluestem (*Schizachyrium scoparium*), is a highly flammable species that plays a crucial role in maintaining sustainable ecosystems and providing important ecosystem services in the eastern portion of the southeastern US Coastal Plain [13–15]. Prescribed burns help stimulate seed production and plant regeneration in savanna understories while also topkilling hardwood trees and maintaining open-canopy conditions [e.g., 16]. Prior to addressing goals for overstory conditions, restoration practitioners strive to establish a self-sustaining grass cover through strategic seeding or planting methods [17].

Efforts to reestablish wiregrass have met with variable success. Both seed source location and competition with neighboring vegetation are drivers of variation in wiregrass survival and growth. Previous research has suggested the existence of locally adapted wiregrass ecotypes, motivating practices for collecting wiregrass seeds on-site or from nearby areas with similar edaphic conditions to those of the restoration site [18, 19]. For example, wiregrass survival may be strongly influenced by matching or contrasting conditions in soil moisture [18, 20]. There is also some evidence that the microbial communities in soils of different moisture capacities affect wiregrass growth [21]. Additionally, planting wiregrass plugs near other vegetation has been observed to reduce survival, suggesting a competitive effect of other native plant species on young plants [9]. Other studies have also indicated that wiregrass survival and growth are sensitive to competition [22, 23]. Once established, however, wiregrass can compete effectively with other species [24, 25], even displaying patterns in density that suggest intraspecific competition [26]. To our knowledge, however, studies have yet to examine the interactive effects of seed source and competition on wiregrass restoration from seed on contrasting soil types.

The objective of this study was to quantify the individual and interactive effects of competition, seed source, and soil type on wiregrass restoration from seed. We hypothesized that the density, size, and flowering culm production of wiregrass plants growing from seed are limited by competition with other plant species. We predicted that wiregrass in plots where competitors were removed would exhibit higher density and larger sizes than those in plots with competing vegetation. Results of our study have important implications for understanding the life history characteristics and increasing restoration success of this foundational species.

## Materials and methods

### Study site and preparation

**Fig 1:**
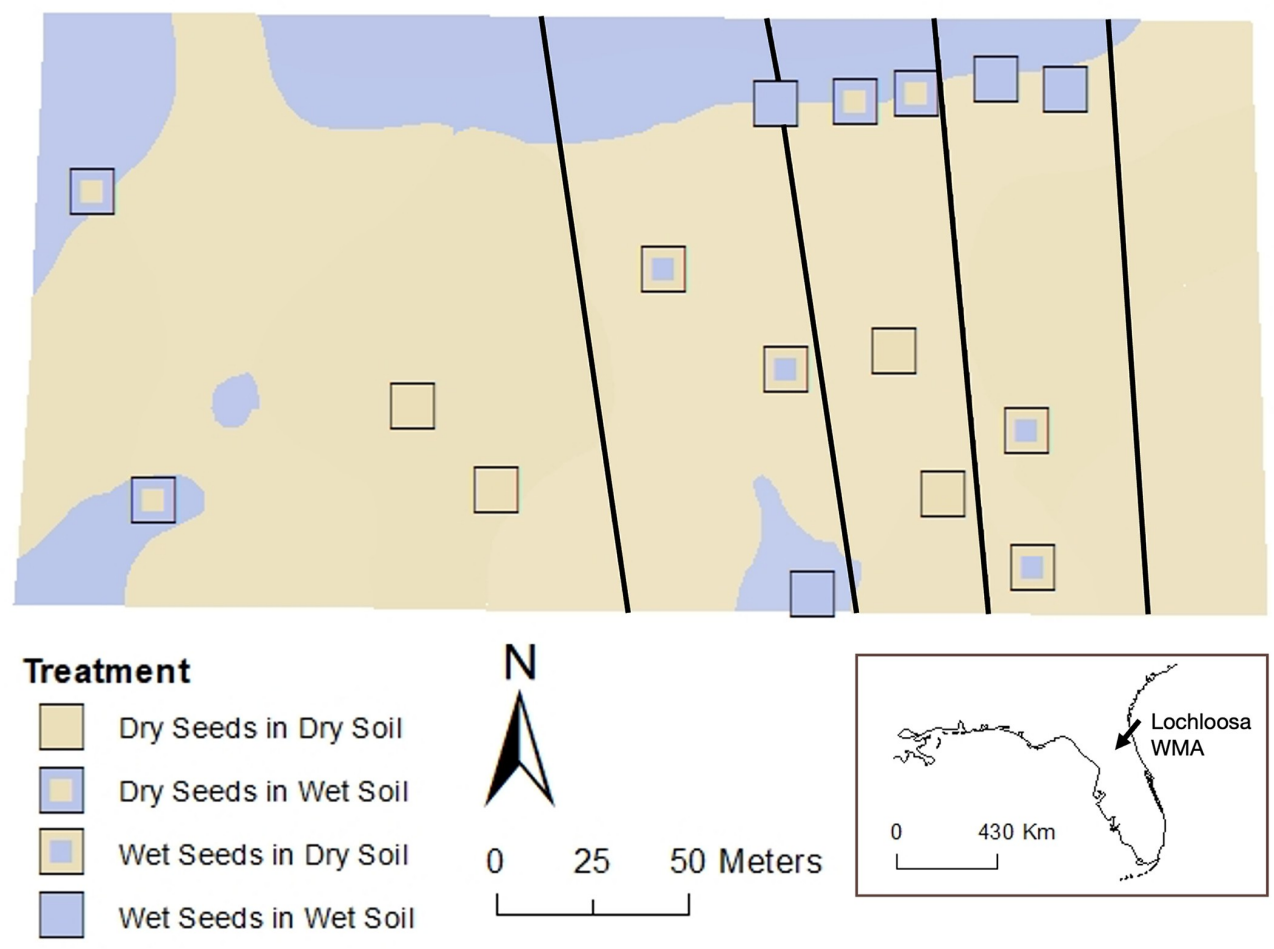
Study site and treatments within Lochloosa Wildlife Management Area (inset) in north-central, Alachua County, Florida. The geographic map was obtained from http://www.naturalearthdata.com/.

For this study, we initiated a 2.0-ha field restoration project (Fig 1) in north-central Florida at the Lochloosa Wildlife Management Area in southeastern Alachua County (29.55231 N, 82.20915 W). This 4451.54-hectare longleaf pine (*Pinus palustris*) flatwoods reserve is jointly managed by the St. Johns River Water Management District and the Florida Fish and Wildlife Conservation Commission. Environments include upland pine, mixed woodland, and sandhills [27], as well as commercial pine plantations and wetlands managed for commercial timber, but the property is slowly being restored to natural pine savannas, beginning with understory wiregrass restoration. Most of the site was cleared in 1937 but reverted to native hardwood secondary growth between 1968 and 1999 when hardwoods were thinned and pines were planted in rows. In 2018, pines were harvested, and the site was mostly cleared except for live oak and southern red oak [28]. The area is characterized by long growing seasons, with an average annual temperature of 20.8°C that varies from 6.7°C to 32.2°C [29]. The dry season lasts 8 months, from September to June, while the wet season lasts 4 months, with an average annual rainfall of 129.54 cm [29].

We used depth to the seasonal water table to delineate areas of “wet” and “dry” soils after assessing field soil cores. Wet soils had a seasonal water table of 0–30 cm below the surface, while dry soils had a water table >30 below the surface, up to 80 cm. In general, our site was covered predominantly by Millhopper (50%; loamy, siliceous, semiactive, hyperthermic Grossarenic Hapludalfs) and Sparr (40%; loamy, siliceous, subactive, hyperthermic Grossarenic Paleudults) fine sands [30]. These soil types have 1–2 meters of sand over an argillic horizon but differ in depth to the seasonal water table. Sparr fine sands tend to have a seasonal high water table from 0.5–1 meter below the surface for 1–4 months every year, while Millhopper has a seasonally high water table between 1–2 meters below the surface. Our site also contained inclusions of the Tavares series (an Aquic Udipsamment [28]), which are somewhat poorly drained and lack an argillic horizon within 2 meters of the surface.

Site preparation for our restoration project began in early September of 2021. Tilling and herbiciding is an important aspect of the restoration process and standard protocol before introducing native groundcover [17]. The site was tilled with a disk harrow in September, and the first herbicide treatments were applied over several days in early October. Herbicide treatments consisted of a 1:100 chemical-water mix per herbicide with surfactant (SilEnergy, Brewer International Inc.,Vero Beach, FL) added to the water at a 1:8 ratio. We used 3.8 liters of triclopyr and 5.7 liters of glyphosate over the whole area. At the end of November 2021, we applied a second treatment of 18.9 liters of glyphosate. A second tilling with the disk harrow took place on December 8, 2021.

The wiregrass seeds used in the experiment were collected during the first week of December. Seeds from wiregrass plants growing in relatively wet environments (hereafter, ‘wet seeds’) were obtained from Hal Scott Preserve, Orange County, FL (~200 km away) in a unit burned on July 15, 2021. We cut inflorescences at the base of the plants and hand-stripped the seeds. Seeds from wiregrass growing on relatively dry soils (hereafter, ‘dry seeds’) were obtained from a site near Econfina Creek Wildlife Management Area, FL (~430 km away, Washington and Bay counties) using a flail-vac in a site burned on April 22, 2021. All seeds were stored in a climate-controlled environment between harvesting and planting. No ethics approval was required for this study.

### Study design

**Fig 2:**
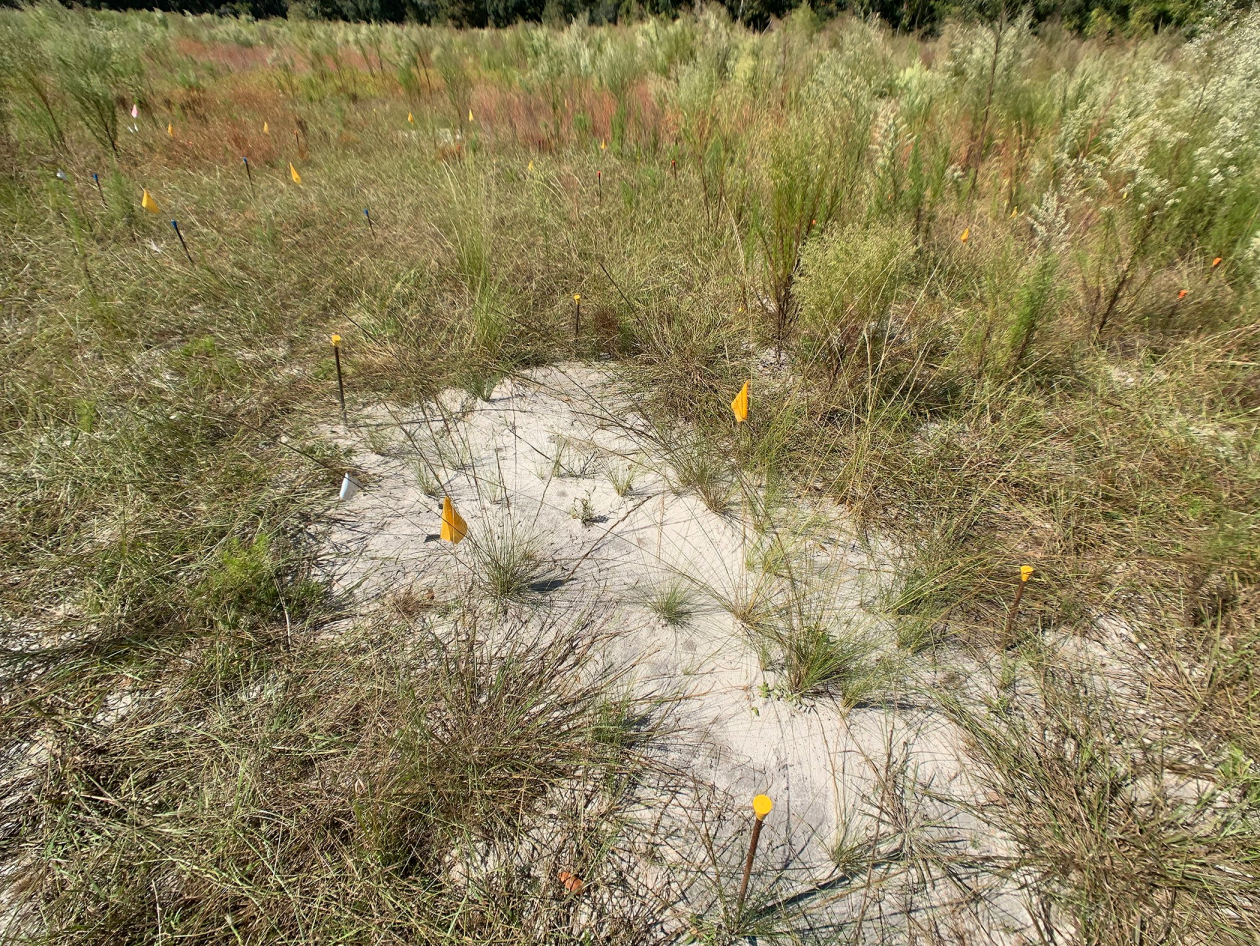
A weeded wiregrass plot, with vegetation removed. In the background is an unweeded plot. Weeded wiregrass plots did not have surface litter removed, and plots varied greatly in the amounts of natural litter present. Yellow flags and rebar caps mark the corners and midpoints of 1×2-m plots. Photo was taken on October 15, 2022.

The wiregrass seeds sourced from dry and wet sites were planted in the field between March 2 and 4, 2022. A Grasslander grass seeder was used for sowing 6.73 kg/ha of wiregrass seeds in four strips running north-south across the field, alternating between dry or wet seeds (Fig 1). We established sixteen groups of eight 1×2-m plots in the field (128 plots total). In each strip, two groups were established on dry soils and two groups were established on wet soils. Four of the eight plots in each group were randomly assigned to be weeded (plant competitors were absent), and four were unweeded (competing vegetation was present; Fig 2). We added wiregrass seeds to each of these plots to increase the sowing rate to 11.21 kg/ha. Plots were recurrently weeded throughout 2022 as necessary from April 22 to May 17, during the week of June 5, and from July 28 to August 10. Non-wiregrass vegetation was removed by pulling, if possible, without disturbing the soil. Otherwise, we clipped the plants at the soil level and removed clippings but left natural litter.

### Data collection

Data on wiregrass plant size, plant number, and number of flowering culms were collected from September 21 to November 18, 2022. The number of wiregrass seedlings in a plot was determined by marking and counting each individual seedling. If seedlings grew close together and appeared to merge into one, they were counted as a single seedling. The perpendicular X and Y width of the plant base (mm) were measured at the soil surface using calipers. Size (basal area) was calculated as the area of an ellipse. Each plant was handled carefully to ensure it was not uprooted or damaged.

### Data analysis

We analyzed the extent to which soil type, seed source, and weeding treatment affected 1) the number of wiregrass plants per plot, 2) the average size of wiregrass plants per plot, 3) the average number of flowering culms per wiregrass plant, and 4) the proportion of flowering wiregrass plants per plot. For the number of culms per plant, we only included flowering plants. All analyses were conducted in R Statistical Software [31].

We fit a negative binomial distribution to the number of wiregrass plants per plot and used ‘glm.nb’ in package *lme4* [32] to run a model with the number of wiregrass plants per plot as the response variable and the main and interactive combinations of seed source, soil type, and weeding treatment as predictor variables. We exponentiated the model coefficients in package *sjPlot* [33] to transform estimates to the original scale (i.e., incidence rate ratios for Poisson models of counts). We log-transformed the wiregrass plant area data and fit a generalized linear regression model with the three-way interaction among seed source, soil type, and weeding treatment as predictor variables using the normal distribution. There were three outliers in the data; the significance of model terms did not change when outliers were removed, so we included them. We log-transformed the average number of culms per plant per plot and fit a generalized linear regression model (‘glm’) with the three-way interaction between seed source, soil type, and weeding treatment as predictor variables, using the normal distribution. We used ‘glmmTMB’ [34] to run a binomial regression on the proportion of flowering plants per plot, with the three-way interaction between seed source, soil type, and weeding treatment and a random term for each individual observation as predictor variables. We exponentiated the model coefficients in package *sjPlot* [33] to transform estimates to the original scale (i.e., odds ratios for binomial models of probabilities or proportions).

## Results

**Fig 3:**
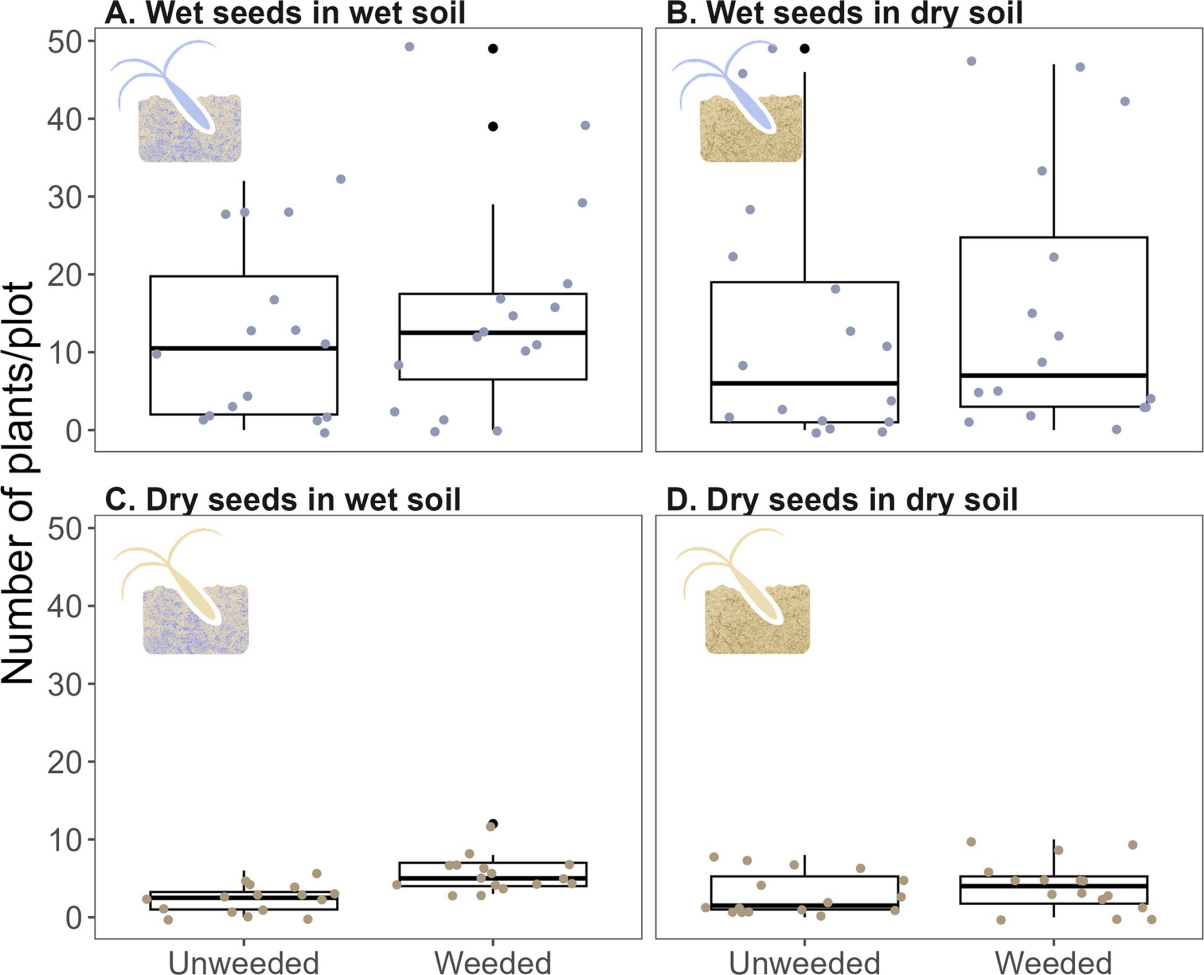
The number of plants in 1×2 m plots for wet seeds in wet soil (A), wet seeds in dry soil (B), dry seeds in wet soil (C), and dry seeds in dry soil. Black points represent outliers, and colored points represent the raw data for each plot.

We found significant effects of seed source and the weeding treatment on our response variables. Our model for the number of wiregrass plants per plot indicated that there were significantly more plants in plots with wet seeds than in plots with dry seeds (Fig 3; Table 1). The median number of plants was 10.5 (range: 0–49) and 3.5 (range: 0–12) in plots with wet and dry seeds, respectively. Plots with wet seeds were likely to have 4.2 times more plants as plots with dry seeds (Table 2). Neither weeding treatment nor soil type significantly affected the number of wiregrass plants per plot. There was also no relationship between the number of wiregrass plants per plot and the average basal area of wiregrass plants per plot (S1 Fig).

**Table 1:** Coefficients, standard errors, and significance values for models of the number of wiregrass plants per plot and the proportion of wiregrass plants with flowering culms per plot.

	Number of wiregrass plants per plot			Proportion of plants with flowering culms per plot		
*Predictor variable*	Estimate	Standard Error	*P*	Estimate	Standard Error	*P*
Intercept	1.12	0.28	**<0.001**	-2.67	0.81	**<0.001**
Weeding Treatment (weeded)	0.30	0.38	0.436	2.22	0.88	**0.012**
Soil Type (wet)	-0.25	0.40	0.522	-0.97	1.33	0.466
Seed Source (wet)	1.44	0.37	**<0.001**	-1.70	1.03	0.099
Weeding Treatment (weeded) x Soil Type (wet)	0.55	0.54	0.308	0.19	1.41	0.893
Weeding Treatment (weeded) × Seed Source (wet)	-0.10	0.52	0.840	1.91	1.12	0.088
Soil Type (wet) × Seed Source (wet)	0.19	0.53	0.720	2.37	1.53	0.122
Weeding Treatment (weeded) × Soil Type (wet) × Seed Source (wet)	-0.52	0.73	0.473	-2.45	1.64	0.135

Predictor variable categories in parentheses are the reference levels for that predictor.

**Table 2:** Ratio values (IRR = Incidence Rate Ratio and OR = Odds Ratio), confidence intervals, and significance values for models of the number of wiregrass plants per plot and the proportion of flowering wiregrass plants per plot.

	Number of wiregrass plants per plot			Proportion of flowering plants per plot		
*Predictor variable*	IRR	CI	*P*	OR	CI	*P*
Intercept	3.06	1.82–5.39	**<0.001**	0.07	0.01–0.34	**0.001**
Weeding Treatment (weeded)	1.35	0.63–2.86	0.436	9.17	1.63–51.62	**0.012**
Soil Type (wet)	0.78	0.35–1.69	0.522	0.38	0.03–5.11	0.466
Seed Source (wet)	4.2	2.04–8.71	**<0.001**	0.18	0.02–1.38	0.099
Weeding Treatment (weeded) x Soil Type (wet)	1.74	0.60–5.06	0.308	1.21	0.08–19.03	0.893
Weeding Treatment (weeded) × Seed Source (wet)	0.90	0.33–2.48	0.840	6.72	0.75–60.19	0.088
Soil Type (wet) × Seed Source (wet)	1.21	0.43–3.41	0.720	10.67	0.53–214.02	0.122
Weeding Treatment (weeded) × Soil Type (wet) × Seed Source (wet)	0.59	0.14–2.48	0.473	0.09	0.00–2.15	0.135

Predictor variable categories in parentheses are the reference levels for that predictor.

**Fig 4:**
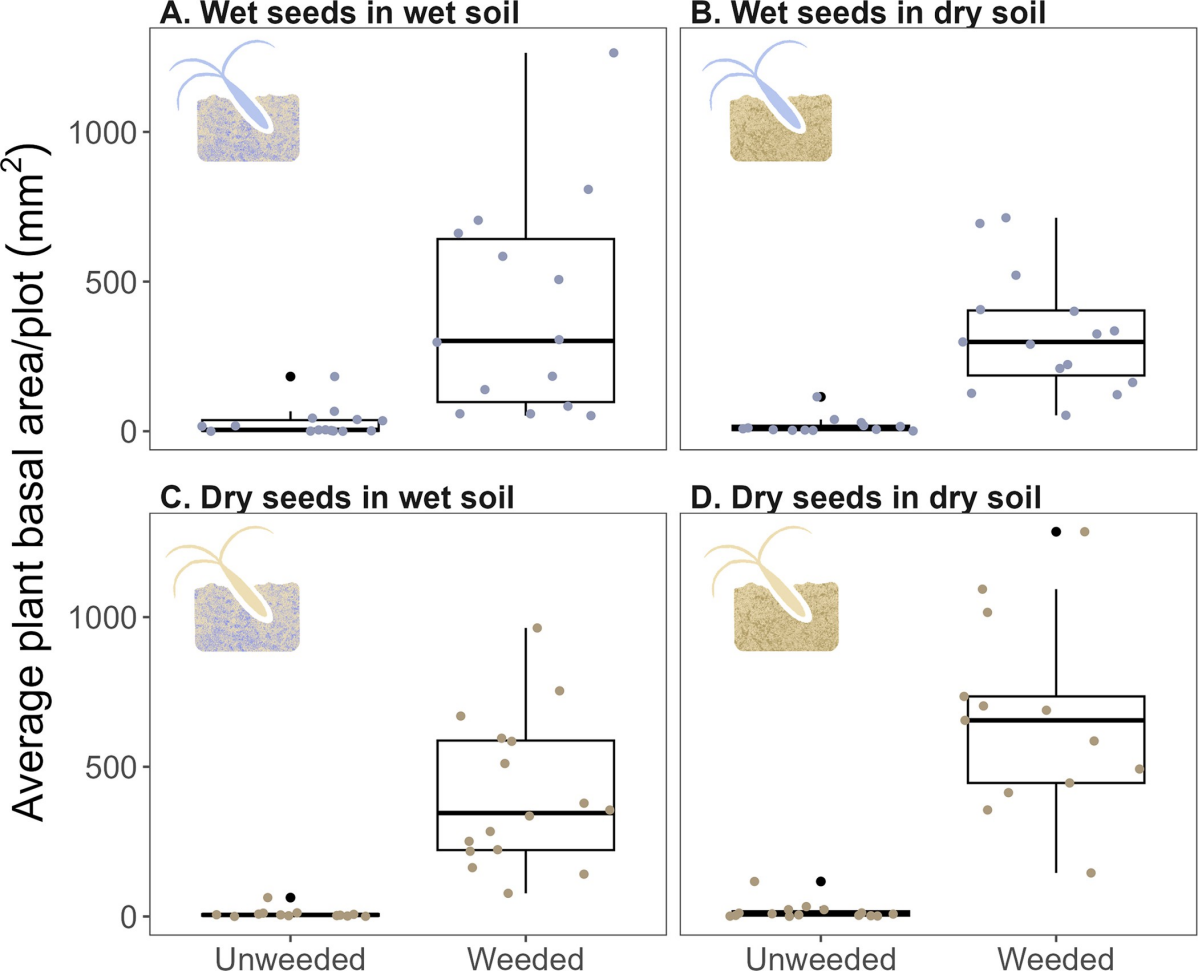
The average basal area of plants in 1×2 m plots for wet seeds in wet soil (A), wet seeds in dry soil (B), dry seeds in wet soil (C), and dry seeds in dry soil. Black points represent outliers, and colored points represent each plot.

The average basal area of plants per plot was significantly greater in the weeded treatment than in the unweeded treatment (Table 3; Fig 4). The average plant basal area per plot ranged from 52 mm² to 1285.3 mm² (median: 367 mm²) in weeded plots and ranged from 0.02 mm² to 182.7 mm² (median: 5.9 mm²) in unweeded plots. The effect of weeding treatment on plant basal area did not differ significantly between seed source or soil type.

**Table 3:** Coefficients, standard errors, and significance values for models of the average basal area of plants per plot (log) and the average number of culms per plant per plot (log).

	Average basal area of plants per plot (log, mm2)			Average number of culms per plant per plot (log)		
*Predictor variable*	Estimate	Standard Error	*P*	Estimate	Standard Error	*P*
Intercept	1.88	0.37	**<0.001**	-0.90	0.63	0.162
Weeding Treatment (weeded)	4.50	0.54	**<0.001**	2.08	0.68	**0.004**
Soil Type (wet)	-0.58	0.54	0.285	-0.90	1.09	0.415
Seed Source (wet)	0.35	0.54	0.515	-1.18	0.89	0.191
Weeding Treatment (weeded) x Soil Type (wet)	0.02	0.75	0.981	-0.02	1.15	0.986
Weeding Treatment (weeded) × Seed Source (wet)	-1.13	0.76	0.138	1.20	0.97	0.219
Soil Type (wet) × Seed Source (wet)	-0.14	0.76	0.854	0.79	1.32	0.555
Weeding Treatment (weeded) × Soil Type (wet) × Seed Source (wet)	0.65	1.06	0.541	0.06	1.43	0.967

Predictor variable categories in parentheses are the reference levels for that predictor.

**Fig 5:**
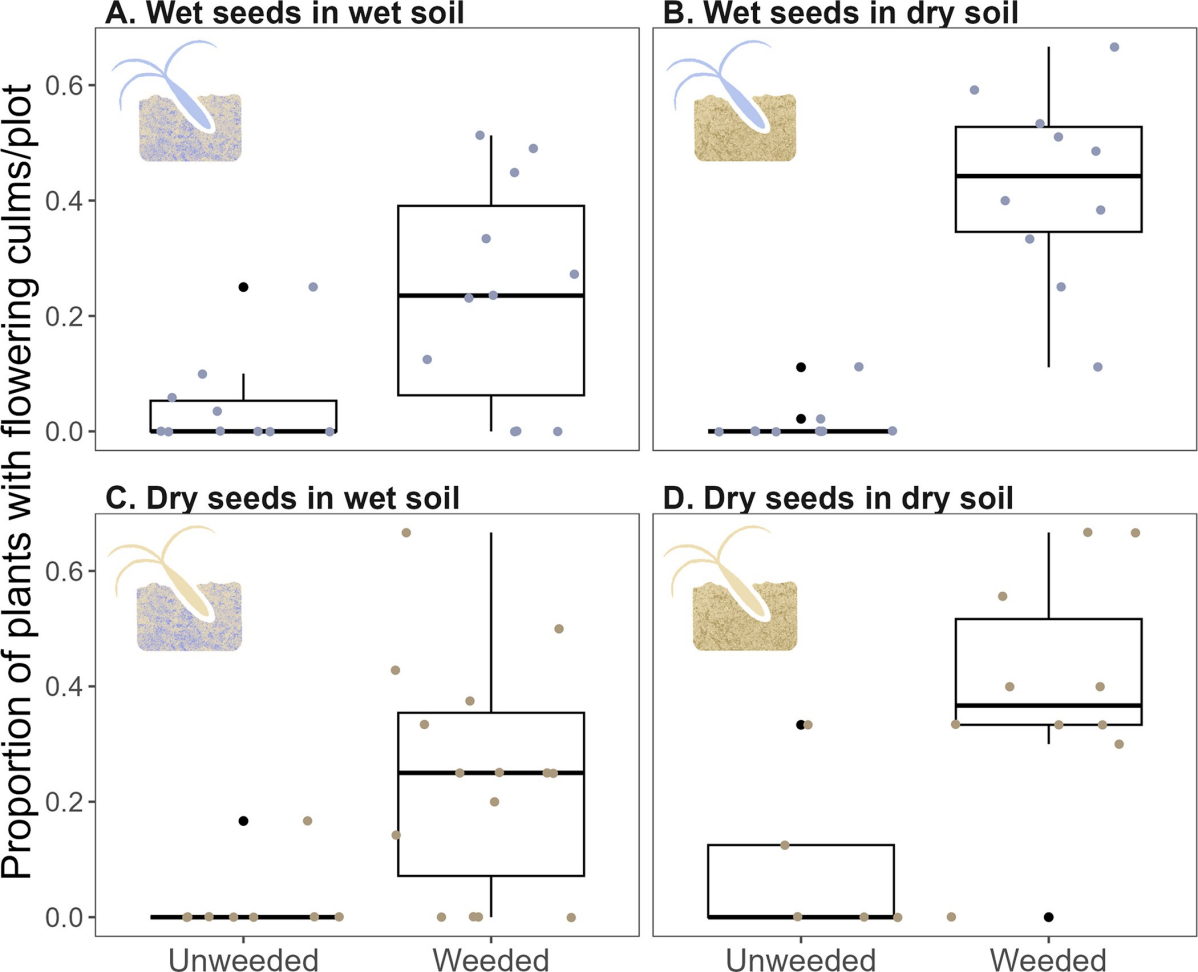
The proportion of plants with flowering culms in 1×2 m plots for wet seeds in wet soil (A), wet seeds in dry soil (B), dry seeds in wet soil (C), and dry seeds in dry soil. Black points represent outliers, and colored points represent each plot.

**Fig 6:**
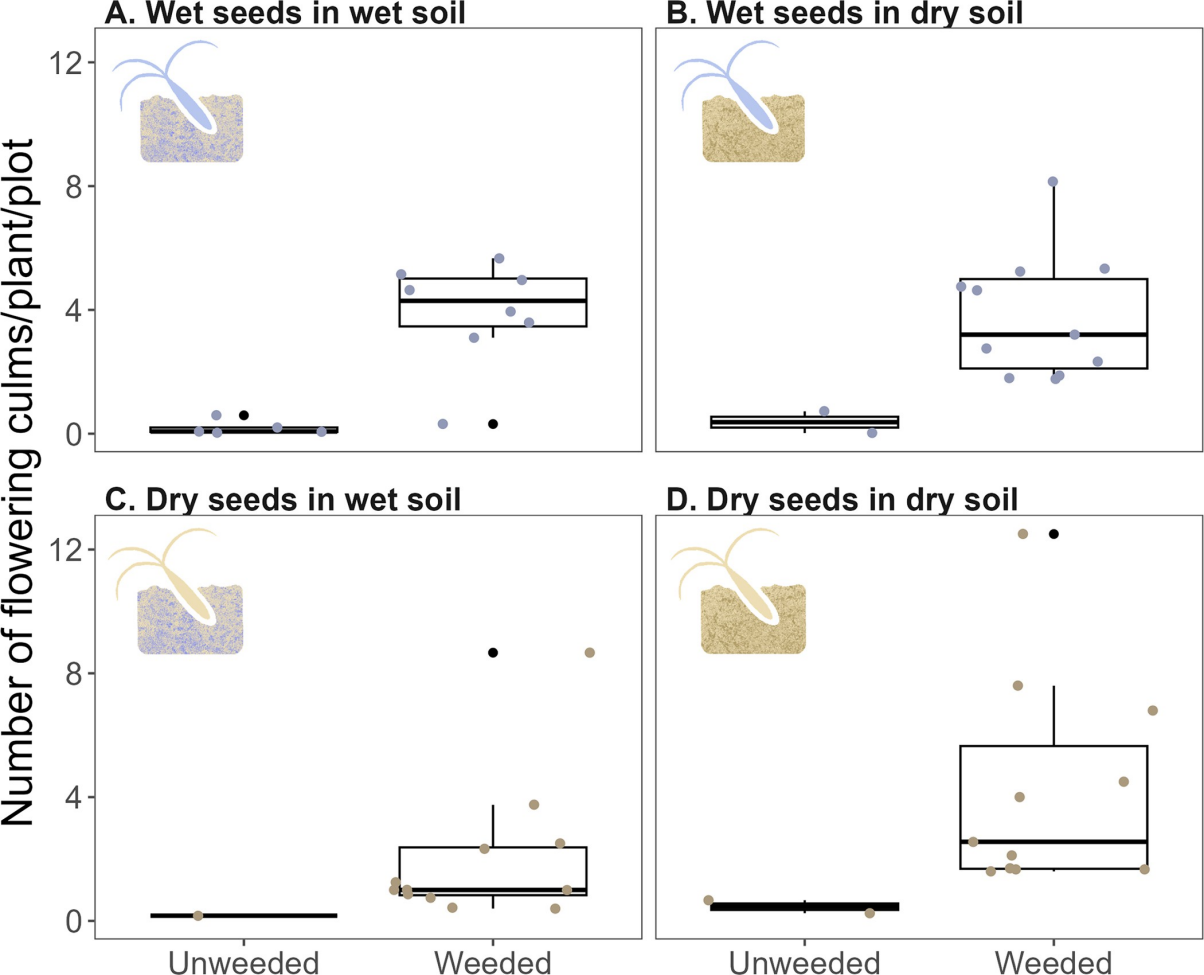
The number of flowering culms for reproducing plants in 1×2 m plots for wet seeds in wet soil (A), wet seeds in dry soil (B), dry seeds in wet soil (C), and dry seeds in dry soil. Black points represent outliers, and colored points represent each plot.

Weeding treatment also affected the production of wiregrass culms. The weeded treatment had a significantly greater average proportion of flowering plants than the unweeded treatment (Tables 1 and 2). The average proportion of flowering plants per plot ranged from 0–0.7 (median: 0.3) and 0–0.3 (median: 0) in weeded and unweeded treatments, respectively. Though the trend was not significant, weeded plots in dry soils had a greater proportion of flowering plants than those in wet soils, while unweeded plots in wet soils had a greater proportion of flowering plants than those in dry soils (Fig 5). In addition, the average number of flowering culms per plant was significantly greater in weeded plots than in unweeded plots (Fig 6; Table 1). The average number of flowering culms per plant ranged from 0.3–12.5 in the weeded treatment (median: 2.7) and from 0.02 to 0.7 (median: 0.2) in the unweeded treatment. The effect of weeding treatment on the average number of flowering culms per plant per plot did not differ between seed source and soil type.

## Discussion

The degree of competition experienced by newly recruiting wiregrass plants likely contributes to variation in wiregrass growth and reproduction across a range of pine savanna sites during the first year of restoration. We found that the presence of competing vegetation significantly affected wiregrass growth and culm production, independent of seed source or soil in which seeds were planted. Although the number of wiregrass plants in each plot did not differ significantly between competition treatments, our wiregrass plants were smaller and had fewer flowering culms in the presence of competing vegetation. Our results correspond to those of [22], who found wiregrass growth to be limited by competition at both three weeks and six months post-germination, although they also found numbers of plants to be reduced by competition at three weeks. Competition affected growth, however, of seedlings of both ages; seedlings were smaller where old field vegetation was present [22]. Their results suggest that competition is less influential on seedling establishment (germination and survival) and more influential on subsequent growth and reproduction. In undisturbed, intact pine savanna communities, on the other hand, competition might have a stronger effect on establishment, given the typically low levels of wiregrass seedling recruitment observed in such communities.

Furthermore, our findings support the need for additional research on factors influencing seed fill and viability. There were no interactive effects of seed source and soil type with or without competition, but seed source significantly affected the number of wiregrass plants in each plot. Wiregrass plants grown from wet seeds were present in higher numbers per plot than those grown from dry seeds, regardless of soil type at the planting location. Based on pre-sowing samples for seed fill, samples of wet seeds were filled at about 55% in contrast to 33% for samples of dry seeds. [35] found that, without controlling for seed fill, the wet seeds had approximately eight times greater viability than the dry seeds. These differences possibly contributed to greater germination and/or higher survival of seedlings from wet seeds. Given that we only collected dry and wet seeds from a single site each, we are unable to draw robust conclusions about whether the difference in numbers of plants is a direct result of seed quality, ecotypic specialization, or a combination of both. Kalmbacher et al. (2004) also found wide variation in seed viability and weather conditions that might have affected their results [20]. At least after the first year, wiregrass is known to tolerate a wide range of pine savanna environments, provided it is planted within acceptable parameters [20].

It should be noted that wiregrass plants rarely flower without being burned, but our results indicate that plants can flower in the first year even without fire. However, we found that the seeds of these plants had extremely low germination (S2 Fig). Although the biological or ecological cues that affect this life history strategy are largely unknown, we can at least include competition among them.

We speculate that the dynamics we observe here may provide support for wiregrass’ role as a foundational species of old-growth savannas. Plant species of mature ecosystems exhibit high competitive ability [36]. Although our young plants under competition were smaller than those in weeded areas, if they survive until mechanisms such as fire release them from competition, they could be considered to exhibit a strategy of slow growth and persistence (e.g., [25]). Regrowth of wiregrass tillers after fire is rapid (authors, unpublished data), and perhaps the growth of young plants is accelerated during the growing season of a fire year. Additionally, the competing vegetation in our study was composed of weedy species that are not typically abundant in old-growth savannas (e.g., dogfennel (*Eupatorium capillifolium*) and yankeeweed (*E*. *compositifolium*)). Once established in natural savannas (e.g., after two years), wiregrass might, in fact, show a strong competitive edge over other perennial plants, greater than that which we observed here.

Seed viability and competition are likely strong drivers of variation in wiregrass restoration outcomes after the first year, regardless of seed sourcing and soil type. Seed viability is a first critical consideration. If seeds do not germinate and survive, there will be no plants to grow and reproduce, and wiregrass seed viability is notoriously variable. A high percentage of wiregrass seeds are often non-viable, and large quantities of seeds are recommended for successful wiregrass restoration [37–39]. After seedlings become established, well into the first year, differing levels of competition contribute to variation in wiregrass growth and reproduction. Although competition removal could help start wiregrass populations, the necessity of mitigation depends on fire management needs. It is common to implement prescribed fire at least two years after planting wiregrass [40]. Spot herbicide treatments applied after sowing could be used to target specific species, but prescribed fire within two years is likely sufficient to control most weedy competitors (K. Madden, personal communication). Removal of weedy species, moreover, could decrease fuel continuity and limit fire spread in the restoration area (K. Madden, personal communication). Given that wiregrass plants were present and reproduced even with weedy competitors present, maintaining continuous fuels to support a frequent fire regime several years after planting likely provides the greatest benefit to wiregrass establishment.

Experimental studies such as ours help reduce uncertainty in the outcomes of restoration approaches. We confirmed competition and seed quality as important drivers of wiregrass restoration results in southeastern US grasslands after the first year. Quantifying the effects of other drivers such as grazing [41], hydrology [42], and soil chemistry [43] can be key in reducing uncertainty in restoration of other grassland systems. Given the wide variability in grassland restoration outcomes over space and time, continued experimental study in different ecosystems provides information that ultimately can assist with identifying common patterns and developing accurate predictive models.

## Supporting information

S1 Fig Relationship between the number of wiregrass (*Aristida beyrichiana*) plants and average plant basal area. Points represent the mean and standard error of each 1 x 2 m plot for wet seeds in wet soil (A), wet seeds in dry soil (B), dry seeds in wet soil (C), and dry seeds in dry soil (D).
(PNG)

S2 Fig The proportion of seeds that germinated, produced by one-year-old wiregrass (*Aristida beyrichiana*) individuals. (PNG)
