# Supplementary figures and images for: Competition limits first-year growth and flowering of wiregrass (Aristida beyrichiana) at a sandhills restoration site
Source: PLoS One. 2024 Sep 3;19(9):e0297795. doi: 10.1371/journal.pone.0297795 (PMC11371212; doi:10.1371/journal.pone.0297795)

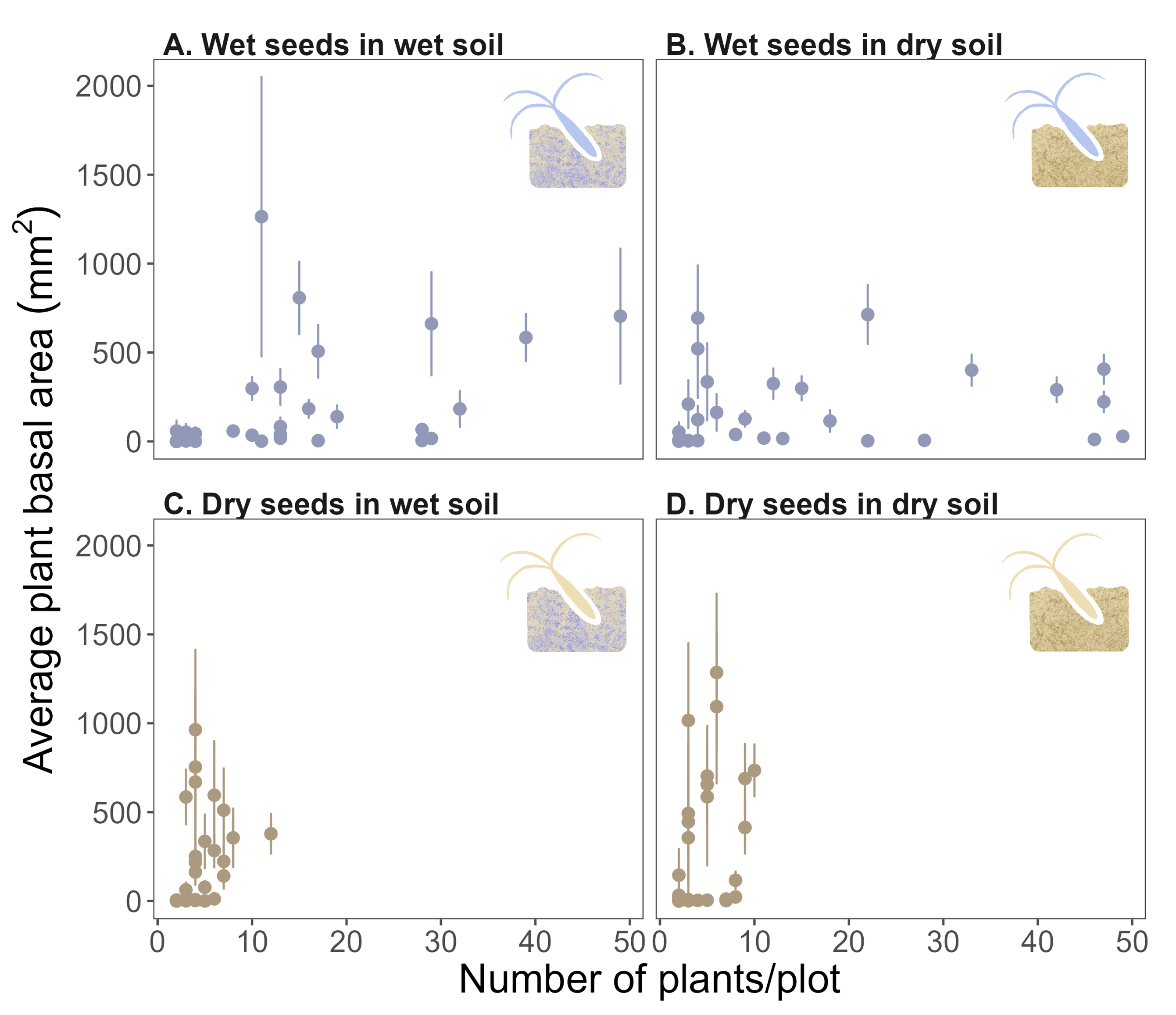

Supplement: S1 Fig — Points represent the mean and standard error of each 1 x 2 m plot for wet seeds in wet soil (A), wet seeds in dry soil (B), dry seeds in wet soil (C), and dry seeds in dry soil (D). (PNG) [file pone.0297795.s001.png]

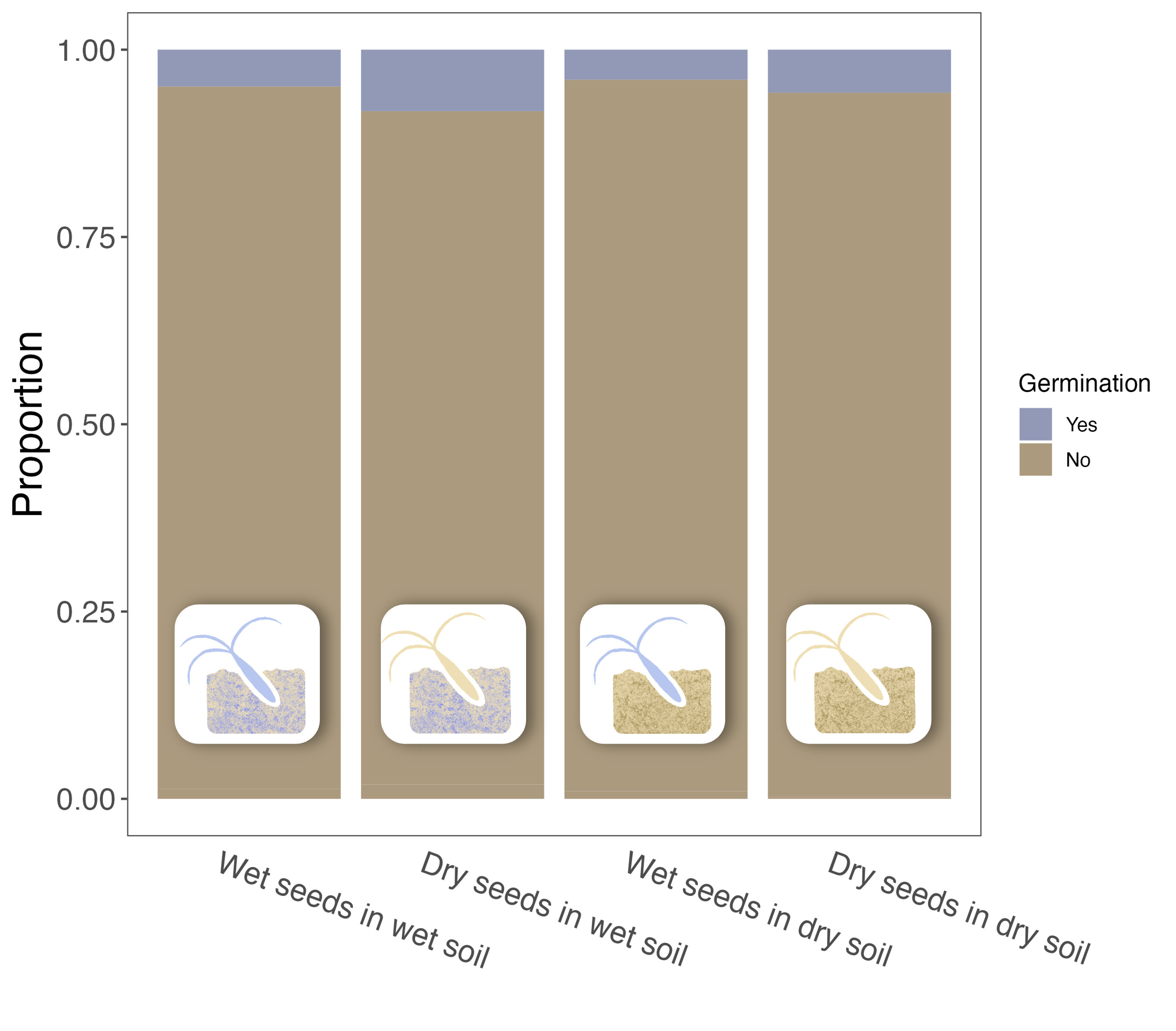

Supplement: S2 Fig — (PNG) [file pone.0297795.s002.png]
